# FDA-approved disulfiram as a novel treatment for aggressive leukemia

**DOI:** 10.1007/s00109-023-02414-4

**Published:** 2024-02-13

**Authors:** Mawar Karsa, Lin Xiao, Emma Ronca, Angelika Bongers, Dayna Spurling, Ayu Karsa, Sandra Cantilena, Anna Mariana, Tim W. Failes, Greg M. Arndt, Laurence C. Cheung, Rishi S. Kotecha, Rosemary Sutton, Richard B. Lock, Owen Williams, Jasper de Boer, Michelle Haber, Murray D. Norris, Michelle J. Henderson, Klaartje Somers

**Affiliations:** 1 Children’s Cancer Institute, Lowy Cancer Research Centre, UNSW Sydney, Sydney, NSW Australia; 2 School of Clinical Medicine, UNSW Medicine & Health, UNSW Sydney, Sydney, NSW Australia; 3 Cancer Section, Development Biology and Cancer Programme, UCL GOS Institute of Child Health, London, UK; 4 ACRF Drug Discovery Centre for Childhood Cancer, Children’s Cancer Institute, Lowy Cancer Research Centre, UNSW Sydney, Sydney, NSW Australia; 5 Leukemia Translational Research Laboratory, Telethon Kids Cancer Centre, Telethon Kids Institute, Perth, WA Australia; 6 Curtin Medical School, Curtin University, Perth, WA Australia; 7 Curtin Health Innovation Research Institute, Curtin University, Perth, WA Australia; 8 Department of Clinical Haematology, Oncology, Blood and Marrow Transplantation, Perth Children’s Hospital, Perth, WA Australia; 9 Division of Paediatrics, School of Medicine, University of Western Australia, Perth, WA Australia; 10 UNSW Centre for Childhood Cancer Research, UNSW Sydney, Sydney, Australia

**Keywords:** Disulfiram, Leukemia, Auranofin, Repurposing, Oxidative stress

## Abstract

**Abstract:**

Acute leukemia continues to be a major cause of death from disease worldwide and current chemotherapeutic agents are associated with significant morbidity in survivors. While better and safer treatments for acute leukemia are urgently needed, standard drug development pipelines are lengthy and drug repurposing therefore provides a promising approach. Our previous evaluation of FDA-approved drugs for their antileukemic activity identified disulfiram, used for the treatment of alcoholism, as a candidate hit compound. This study assessed the biological effects of disulfiram on leukemia cells and evaluated its potential as a treatment strategy. We found that disulfiram inhibits the viability of a diverse panel of acute lymphoblastic and myeloid leukemia cell lines (*n* = 16) and patient-derived xenograft cells from patients with poor outcome and treatment-resistant disease (*n* = 15). The drug induced oxidative stress and apoptosis in leukemia cells within hours of treatment and was able to potentiate the effects of daunorubicin, etoposide, topotecan, cytarabine, and mitoxantrone chemotherapy. Upon combining disulfiram with auranofin, a drug approved for the treatment of rheumatoid arthritis that was previously shown to exert antileukemic effects, strong and consistent synergy was observed across a diverse panel of acute leukemia cell lines, the mechanism of which was based on enhanced ROS induction. Acute leukemia cells were more sensitive to the cytotoxic activity of disulfiram than solid cancer cell lines and non-malignant cells. While disulfiram is currently under investigation in clinical trials for solid cancers, this study provides evidence for the potential of disulfiram for acute leukemia treatment.

**Key messages:**

- Disulfiram induces rapid apoptosis in leukemia cells by boosting oxidative stress.
- Disulfiram inhibits leukemia cell growth more potently than solid cancer cell growth.
- Disulfiram can enhance the antileukemic efficacy of chemotherapies.
- Disulfiram strongly synergises with auranofin in killing acute leukemia cells by ROS induction.
- We propose testing of disulfiram in clinical trial for patients with acute leukemia.

**Supplementary Information:**

The online version contains supplementary material available at 10.1007/s00109-023-02414-4.

## Introduction

Acute leukemia remains a fatal cancer in most adults and one of the most common causes of death from disease in children [1–4]. Survival rates of patients suffering from high-risk subtypes including *KMT2A*-r rearranged (*KMT2A*-r) leukemia and relapsed T cell acute lymphoblastic leukemia (ALL) remain below 50% [1, 5]. Currently applied chemotherapeutics have reached their limit of tolerability, leading to an increased focus on identifying novel treatment approaches such as molecularly targeted therapies and new drug combinations to increase treatment efficacy and circumvent resistance. However, the trajectory for the development of new drugs is a time-consuming, expensive, and risky process with a high failure rate. Drug repurposing, in which drugs are used for the treatment of diseases for which they were not originally developed, can represent an alternate and more efficient drug development pipeline to allow for more rapid advancement of therapies for clinical use [6].

We previously employed a drug repurposing approach in which we screened drug libraries composed of FDA-approved drugs and bioactive compounds to identify agents that could be repositioned for the treatment of acute leukemia [7, 8]. A first screen encompassed a phenotypic, cell-based viability screen with cell lines derived from children with high-risk leukemia, including infants diagnosed under 1 year of age with *KMT2A*-r leukemia, and relapsed T-cell ALL (T-ALL) [7]. A second independently executed study was designed to specifically address the urgent need for novel therapeutics for *KMT2A*-r leukemias and entailed a high-throughput screen for drugs that specifically deplete oncogenic MLL fusion proteins, using a reporter cell line for MLL fusion protein expression [8]. Both screens independently identified disulfiram as a candidate antileukemic agent [7, 8].

Disulfiram, or Antabuse, is approved to support the treatment of chronic alcoholism. The drug targets the enzyme acetaldehyde dehydrogenase, inhibiting the normal breakdown of acetaldehyde downstream of alcohol catabolism by the liver, which causes a ‘hangover’ effect immediately following alcohol consumption [9]. The potential of disulfiram to exert anticancer effects was first noted in 1977, when a patient with metastatic breast cancer who was treated with disulfiram to manage their alcoholism, went into remission without receiving any other treatment [10]. Since then, several preclinical studies have reported on the anticancer activity of disulfiram, culminating into its testing in clinical trials for several solid cancers including melanoma, glioblastoma, pancreatic and breast cancer [11].

Here, we further explore the biological and therapeutic effects of disulfiram on childhood and adult acute leukemia cells and provide preclinical evidence that supports investigation of the drug’s potential to treat acute leukemia in the clinical setting.

## Materials and methods

### Cell lines and patient-derived xenograft cells

The panels of cell lines and patient-derived xenograft (PDX) cells used in this study are described in Supplementary Tables 1 and 2 [12]. All cell lines were authenticated within the last 3 years and all experiments were performed with mycoplasma-free cells.

### Cytotoxicity and synergy assays

The cytotoxicity of disulfiram was assessed by performing resazurin reduction-based viability assays with acute leukemia cell lines and PDX cells as previously described [13, 14]. The values of the inhibitory concentration resulting in a 50% reduction of cell viability relative to control (IC_50_) were calculated by non-linear regression. Co-culture experiments with human telomerase reverse transcriptase (hTERT)-immortalized human mesenchymal stromal cells (hTERT-MSC) were performed as described previously [15]. Briefly, hTERT-MSCs were seeded (200,000/well) in culture medium in 24-well plates and left overnight to form adherent monolayers. Cells were subsequently transferred to hTERT-MSC-lined wells, followed by addition of disulfiram to achieve a final concentration of 200 nM. Following a 24h drug exposure, the proportion of dead cells was determined by 7-AAD exclusion using a FACSCanto II flow cytometer.

Synergy studies were performed in fixed ratio or 6 × 6 matrix assays [7] and synergy was assessed with the Bliss Independence model as previously described [7, 16]. Bliss Prediction curves reflect the predicted % viability of the cells when exposed to the combination of compounds if both compounds are additive. Synergy is defined when a lower cell viability upon combination of two compounds is measured compared to the viability predicted based on the presence of an additive effect of the compounds (i.e., the viability curve of the cells treated with the drug combination runs below the Bliss prediction curve). Excess over Bliss (EOB) values were obtained by calculating the difference between the experimentally observed fraction of cells affected by the drug combination and the expected fractional inhibition of the drug combination in case of an additive drug interaction [16, 17]. Individual EOB values across all concentration combination points used in the combination assays were summed to calculate the EOB Sum of the combination [16, 17]. An EOB value of 0 corresponds to an additive effect while EOB values greater or below 0 represent synergism or antagonism, respectively. Synergy determined in 6 × 6 matrix assays was visualized through Combenefit [18].

### Apoptosis assays

The percentage of apoptotic cells was determined by staining with Annexin V and 7-Aminoactinomycin D (7-AAD) (BD Biosciences, Australia) followed by flow cytometry detection as previously described [13].

### Reactive oxygen species assays

Reactive oxygen species (ROS) levels were determined by flow cytometry. Treated cells were stained with 2′,7′-dichlorofluorescin diacetate (DCFDA) (Sigma-Aldrich, Australia) for 1 h or MITOSOX™ Red Mitochondrial Superoxide Indicator (Thermo Fisher Scientific, Australia) for 15 min in the dark, according to manufacturer’s instructions. Samples were analyzed on a FACSCalibur (BD Biosciences, Australia). FlowJo (Becton, Dickinson & Company, USA) was used to determine the mean fluorescence intensity (MFI) of treated samples relative to control cells. For rescue experiments, cells were pre-treated with 5 mM *N*-acetyl cysteine (NAC) for 1 h prior to disulfiram treatment.

### Immunoblotting

Protein analysis and immunoblotting were performed as previously described [13, 14]. The following primary antibodies were used: anti-β-ACTIN Sigma-Aldrich (Castle Hill, NSW, AUS), anti-NRF2 (Merck KGaA, Darmstadt, Germany), and antibodies against phospho-histone H2A.X, cleaved PARP and HMOX1 (Cell Signaling Technology, Inc., MA, USA).

### Quantitative RT-PCR for KMT2A target genes

Expression levels of *HOXA10*, *MEIS1*, and *MYB* were determined as previously described [8, 13].

### Statistical analyses

GraphPad Prism 9 (GraphPad software, San Diego, CA, USA) was used for all analyses. One sample *t* tests or Student’s *t* tests were used to assess statistical significance of differences in measurements between two groups. One-way ANOVA with Tukey’s correction for multiple comparisons was employed when analyzing significance involving three or more groups. *P* values less than 0.05 were considered statistically significant.

## Results and discussion

### Disulfiram is more cytotoxic to acute leukemia cell lines than to solid tumor cell lines

In our previously executed repurposing screening campaign conducted in cell lines derived from children with high-risk leukemias, disulfiram was one of ten hit compounds that more potently decreased the viability of leukemia cell lines than of non-malignant cells [7]. Disulfiram also constituted one of three agents that reduced leukemogenic MLL-AF9 fusion protein levels in reporter leukemia cells by more than 33% [8].

**Fig. 1:**
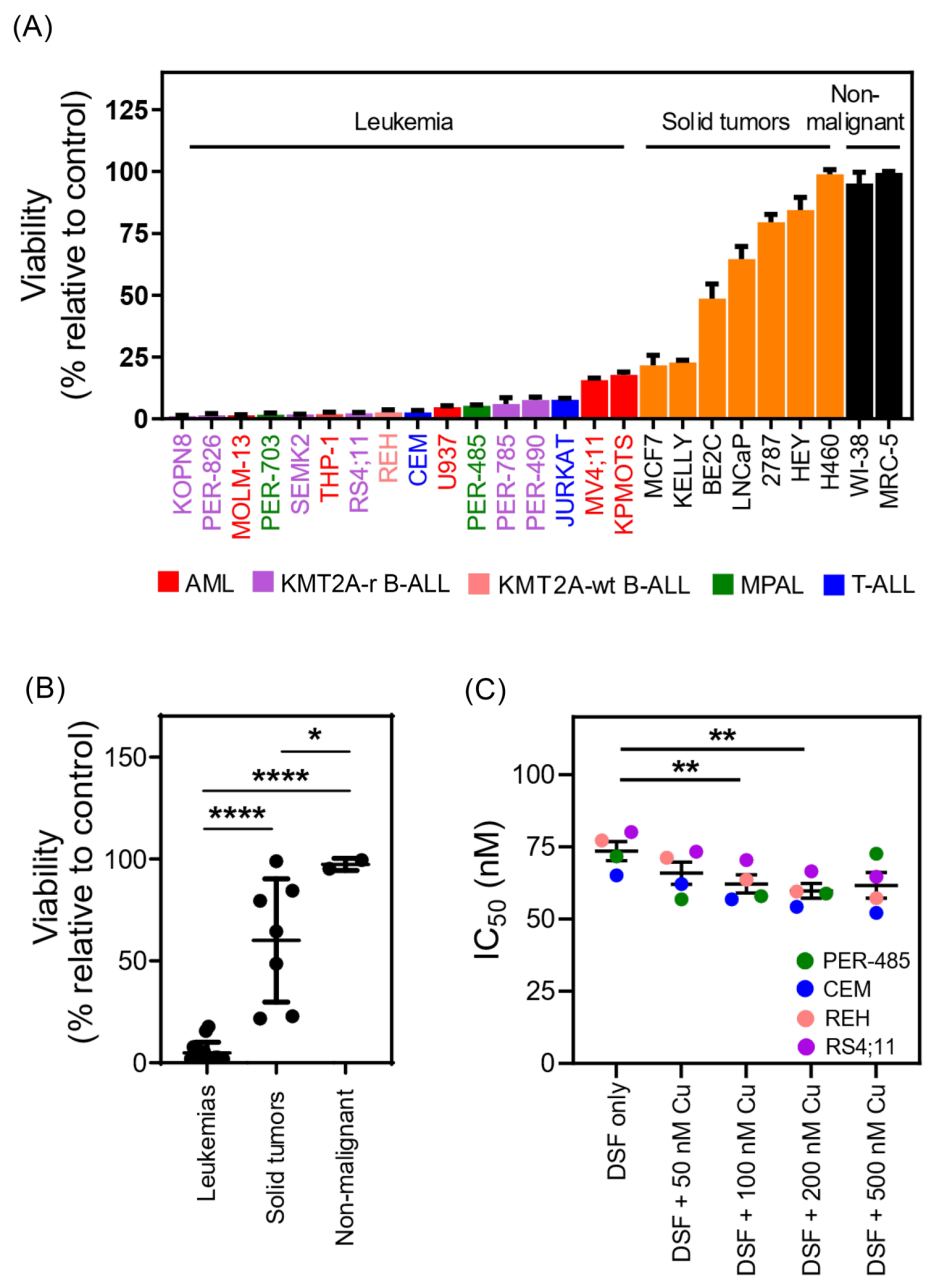
Disulfiram exerts preferential cytotoxic activity against acute leukemia cells. **A** Viability of a cell line panel comprising acute leukemia, solid tumor, and non-malignant cell lines after treatment with 100 nM of disulfiram (DSF) for 72 h, as measured by resazurin reduction-based cytotoxicity assays. Viability percentages are expressed relative to control cells. Mean viability percentages ± SE from three independent experiments are shown. **B** Comparison of the cell viability of leukemia, solid tumor, and non-malignant cell lines after treatment with 100 nM DSF for 72 h. Dots represent mean viability of three replicates. Mean viability percentages after treatment with 100 nM DSF were compared between groups by one-way ANOVA with Dunn’s correction for multiple comparisons. **C** Sensitivity of leukemia cell lines treated with DSF alone and in combination with 50 nM, 100 nM, 200 nM, or 500 nM of copper (Cu). The significance of the difference in mean IC_50_ values between the groups treated with DSF + Cu compared to DSF alone was determined by One-way ANOVA with Tukey's corrections for multiple comparisons. Asterisks represent *p* values: *p < 0.05; **p < 0.01; ****p < 0.0001

As these studies implicated disulfiram as a potential antileukemia drug, we therefore undertook full dose–response resazurin reduction-based viability assays to thoroughly characterize the effects of the drug in a larger and more diverse panel of cells comprised of acute leukemia cell lines (*n* = 16), solid cancer cell lines (*n* = 7), and non-malignant cells (*n* = 2). The 16 leukemia cell lines were derived from patients with high-risk disease including infants with *KMT2A*-r leukemia, one of the poorest outcome pediatric leukemias, T-ALL, mixed phenotype acute leukemia (MPAL), and *CALM-AF10* translocated AML (Supplementary Table 1). Disulfiram decreased the viability of all leukemia cell lines with IC_50_ values ranging from 45 to 81 nM, and 100 nM of the drug decreased the viability of all leukemia cell lines to below 20% (Fig. 1A, Supplementary Fig. 1, Table 1). While a trend was observed for higher IC_50_ values for AML/MPAL cells, no significant associations were observed between disulfiram sensitivity and leukemia subtype based on immunophenotype (AML/MPAL versus B-ALL versus T-ALL) or molecular or genetic aberrations (Supplementary Fig. 2), demonstrating the pan-antileukemia potential of the drug. Interestingly, acute leukemia cell lines were significantly more sensitive to disulfiram than cell lines derived from solid cancers (*p* < 0.0001) or non-malignant (*p* < 0.0001) cells (Fig. 1B) suggesting an increased susceptibility of acute leukemia cells to the cytotoxic effects of the drug. This is supported by prior findings that disulfiram does not negatively impact human CD34^+^ cord blood cells in colony formation assays at concentrations that significantly abrogate colony formation capability of *KMT2A*-r leukemia cells [8].

**Table 1:** Sensitivity of acute leukemia, solid tumor, and non-malignant cell lines to disulfiram

**Cell lines**			**Disulfiram IC**_**50**_ **(nM)**
**Leukemia cells**			
T-ALL	CCRF-CEM (CEM)		61
	Jurkat		58
B-ALL	PER-826	*KMT2A*-r	45
	PER-490	*KMT2A*-r	56
	PER-785	*KMT2A*-r	49
	KOPN-8	*KMT2A*-r	45
	RS4;11	*KMT2A*-r	46
	SEMK2	*KMT2A*-r	49
	REH		52
MPAL	PER-485	*KMT2A*-r	60
	PER-703	*KMT2A*-r	47
AML	THP-1	*KMT2A*-r	51
	MV4;11	*KMT2A*-r	57
	MOLM13	*KMT2A*-r	63
	U937		65
	KP-MO-TS		81
**Solid tumor cells**			
	KELLY	Neuroblastoma	73
	BE(2)-C	Neuroblastoma	81
	HEY	Ovarian cancer	115
	2787	Endometrial ovarian cancer	49
	MCF-7	Breast adenocarcinoma	> 1000
	H460	Lung carcinoma	500
	LNCaP	Prostate carcinoma	106
**Non-malignant cells**			
	MRC-5	Lung fibroblast	> 1000
	WI-38	Lung fibroblast	> 1000

As previous studies on disulfiram reported that the drug’s ability to chelate copper contributes to its anticancer properties [19], we tested whether the addition of copper boosted the cytotoxic activity of disulfiram against a diverse panel of leukemia cell lines derived from different leukemia subtypes, namely PER-485 (MPAL), RS4;11 (*KMT2A*-r B-ALL), REH (B-ALL) and CEM (T-ALL) cells. The addition of copper significantly decreased the IC_50_ values of disulfiram against acute leukemia cells, slightly increasing the antileukemic activity of the drug (Fig. 1C, Supplementary Fig. 3A). However, the supplementation of copper also increased the sensitivity of solid tumor cells and more importantly non-malignant MRC-5 cells to disulfiram, suggesting a potentially narrower therapeutic window for the disulfiram/copper combination than for disulfiram alone (Supplementary Fig. 3B). These findings point toward the need for a careful evaluation of the clinical effects of the addition of supplementary copper to disulfiram upon testing in patients.

### Disulfiram induces rapid apoptosis in acute leukemia cells by causing oxidative stress

**Fig. 2:**
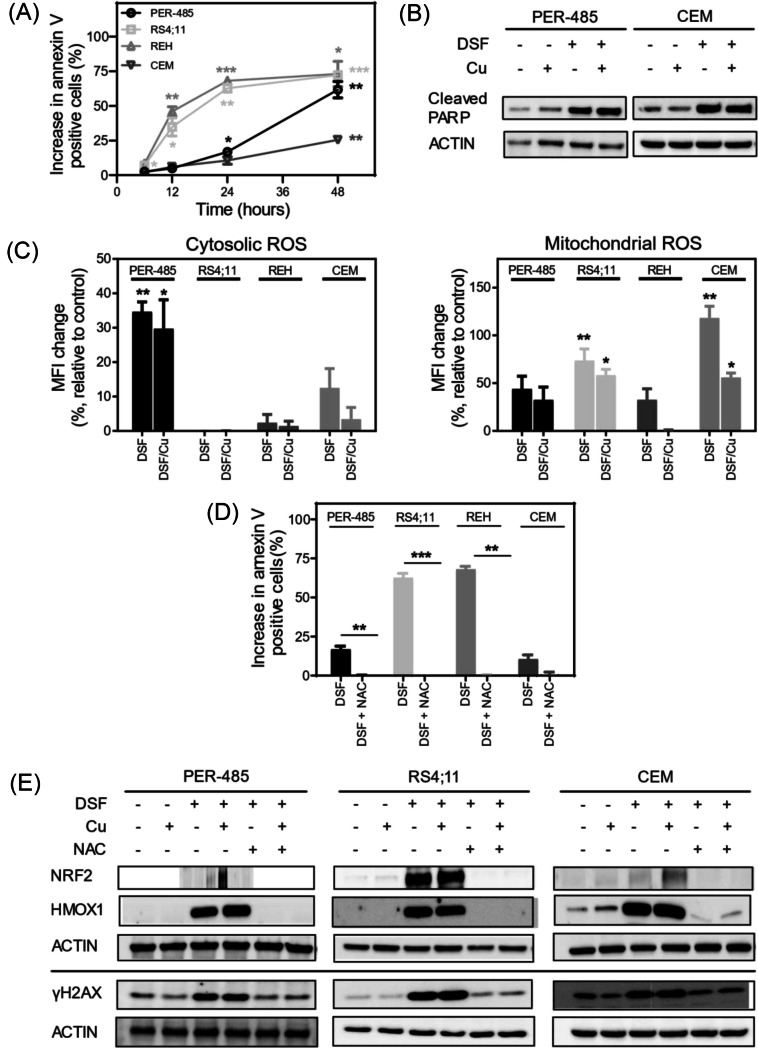
Disulfiram kills leukemia cells by inducing oxidative stress. **A** Flow cytometric analysis of annexin V staining after treatment with 60 nM disulfiram (DSF) for 6 to 48 h. Apoptosis is expressed as the percentage increase in annexin V-positive cells relative to untreated controls. Results are expressed as the mean ± SE of at least two independent experiments and for each cell line the significance of the mean percentage increase relative to untreated controls is determined by one sample *t* tests for each time point. **B** Immunoblot of cleaved PARP following a 6 h treatment of cells with 60 nM DSF, with and without 100 nM copper (Cu). Total actin was used as a loading control. **C** Cytosolic and mitochondrial reactive oxygen species (ROS) levels as measured by flow cytometric analysis of 2′,7′-dichlorofluorescin diacetate (DCFDA) and MitoSOX, respectively, after treatment with 100 nM DSF for 6 h, in the presence and absence of 100 nM Cu. ROS levels are expressed as the percentage change in mean fluorescence intensity (MFI) relative to untreated cells. The bars represent mean values from at least two independent experiments. Mean MFI changes were compared by one-way ANOVA with Tukey’s correction for multiple comparisons. **D** Impact of *N*-acetyl cysteine (NAC) pretreatment on apoptosis induced by DSF as measured by annexin V/7-AAD staining. The apoptosis results are expressed as the mean ± SE of three independent experiments. Group means between DSF and NAC + DSF treated cells were compared by paired *t* tests. **E** Representative immunoblot for NRF2, HMOX1, and γH2AX after treatment with DSF (60 nM) for 6 h in the presence and absence of 100 nM Cu. Total actin was used as a loading control. Western blotting was independently performed at least twice. Asterisks represent significance levels of p-values. *p < 0.05; **p < 0.01; ***p < 0.001

The potential of disulfiram to induce apoptosis as a mechanism to limit leukemia cell viability was measured by assessing 7AAD/annexin V staining of cells exposed to the drug for up to 48 h. Disulfiram rapidly increased the percentage of cells staining positive for annexin V in a time-dependent manner, providing evidence for the drug inducing apoptosis in leukemia cells within 12 h of drug exposure (Fig. 2A). Disulfiram treatment increased the levels of cleaved PARP, a marker of apoptosis, in leukemia cells with or without copper, further confirming the apoptosis-inducing effect of the drug (Fig. 2B). Similar to our findings in liquid cultures, disulfiram treatment significantly increased the proportion of dead leukemia cells in a human mesenchymal stem cell (MSC) co-culture system, while adherent solid tumor-derived cells (H460) and non-malignant cells (MRC-5) were unaffected in these assays (Supplementary Fig. 4).

Several potential anticancer mechanisms have been attributed to disulfiram including proteasome inhibition, augmented production of ROS, and activation of the MAPKinase pathway [20–22]. Based on our previously reported finding that leukemia cells have significantly lower glutathione content and higher ROS levels than solid tumor cell lines [7], and our current observation that leukemia cell lines are more sensitive to disulfiram than cell lines derived from solid tumors (Fig. 1B), we hypothesized that an important antileukemic mechanism of the drug is to boost the generation of ROS in leukemia cells, thereby tipping the oxido-reductive balance to induce oxidative stress and subsequently apoptosis. To address this hypothesis, we assessed the level of cytosolic and mitochondrial ROS in leukemia cells after treatment with disulfiram. Indeed, disulfiram significantly increased the levels of either cytosolic or mitochondrial ROS in three out of four cell lines (PER-485 [23], RS4;11 and CEM cells) within 6 h, while a trend for increased mitochondrial ROS levels was observed in REH cells (Fig. 2C). The presence of copper did not further augment ROS levels at this early time point (Fig. 2C). Pre-treatment with a ROS scavenger, NAC, prevented ROS accumulation (Supplementary Fig. 5) and completely abolished the induction of apoptosis (Fig. 2D), indicating that disulfiram-mediated cell death is largely caused by oxidative stress. In further support of this finding, the drug induced expression of several oxidative stress pathway proteins, including nuclear factor (erythroid-derived 2)-like 2 (NRF2), a master regulator of the antioxidant response and haem oxygenase 1 (HMOX1), encoded by the NRF2 target gene *HMOX1* (Fig. 2E). The increased expression of NRF2 and HMOX1 was abrogated by pre-treatment with NAC (Fig. 2E). High levels of oxidative stress limit the viability of cancer cells by inducing damage to DNA, RNA, proteins, lipids, and mitochondria. Disulfiram, both in the absence and presence of copper, increased expression of phosphorylated H2A histone family, member X (γH2AX), a marker for DNA damage. Pre-treatment with NAC rescued the induction of DNA damage (Fig. 2E), indicating that the DNA damage accumulation is a downstream effect of disulfiram-mediated oxidative stress.

We previously reported that disulfiram inhibits the functioning of MLL fusion oncoproteins in *KMT2A*-r leukemia [8]. This highly aggressive and treatment-resistant leukemia subtype is driven by a chromosomal rearrangement between the *KMT2A* gene encoding MLL1, a histone methyl transferase, and one of over 100 different translocation partners [24, 25]. The chromosomal translocation generates a chimeric MLL fusion protein that causes aberrant expression of a large collection of normally tightly regulated genes (e.g., *HOX* genes, *MEIS1*, *MYB*), which has been shown to be leukemogenic [26]. We previously found that disulfiram prevents MLL fusion protein binding to DNA in *KMT2A*-r leukemia cells including MV4;11 cells [8]. To investigate whether disulfiram exerts part of its cytotoxic effects on *KMT2A*-r leukemia cells by this action, we evaluated whether the drug inhibited the expression of MLL fusion protein target genes prior to ROS and apoptosis induction. We therefore assessed the expression of target genes *HOXA10*, *MEIS1*, and *MYB* in *KMT2A*-r leukemia cells MV4;11 and PER-485 after a short (4h) treatment with disulfiram. Although disulfiram significantly decreased expression of several MLL fusion protein target genes within a few hours of treatment, prior to ROS induction, the inhibition was only partial and variable across cell lines (Supplementary Fig. 6). It is unclear whether the observed changes in target gene expression would significantly impact *KMT2A*-r leukemia cell viability. While it is possible that disulfiram is able to reverse the leukemogenic gene signature induced by MLL fusion proteins in *KMT2A*-r leukemia cells preceding ROS induction and apoptosis, our data support that in liquid in vitro culture, ROS induction is largely responsible for the cell death induced by disulfiram in *KMT2A*-r leukemia cells. Notwithstanding, it is possible that the mechanism might contribute to the antileukemic effects of disulfiram in other settings (e.g., in vivo). Moreover, as several studies indicate a role for MLL fusion target genes including *MEIS1* in regulating oxidative stress, it is possible that decreased MLL fusion target gene expression even contributes to some extent to tipping the oxidative stress balance in *KMT2A*-r leukemia cells upon disulfiram treatment [27–29].

Taken together, our data indicate that the main mechanism by which disulfiram induces apoptosis in leukemia cells is by augmenting ROS levels and triggering oxidative stress. However, as disulfiram affects many cellular pathways, this is unlikely to be the complete story [11]. Many other anticancer mechanisms have previously been observed for disulfiram and the drug’s mechanism of action is likely to be multi-modal and dependent on the molecular wiring of the cancer cell [11]. This characteristic of disulfiram could be valuable in view of the intra- and inter-tumoral heterogeneity within and between patients and in light of the known risks of developing therapy-resistance when targeting only a single cancer cell pathway [30].

### Disulfiram synergizes with standard of care chemotherapies and auranofin

**Fig. 3:**
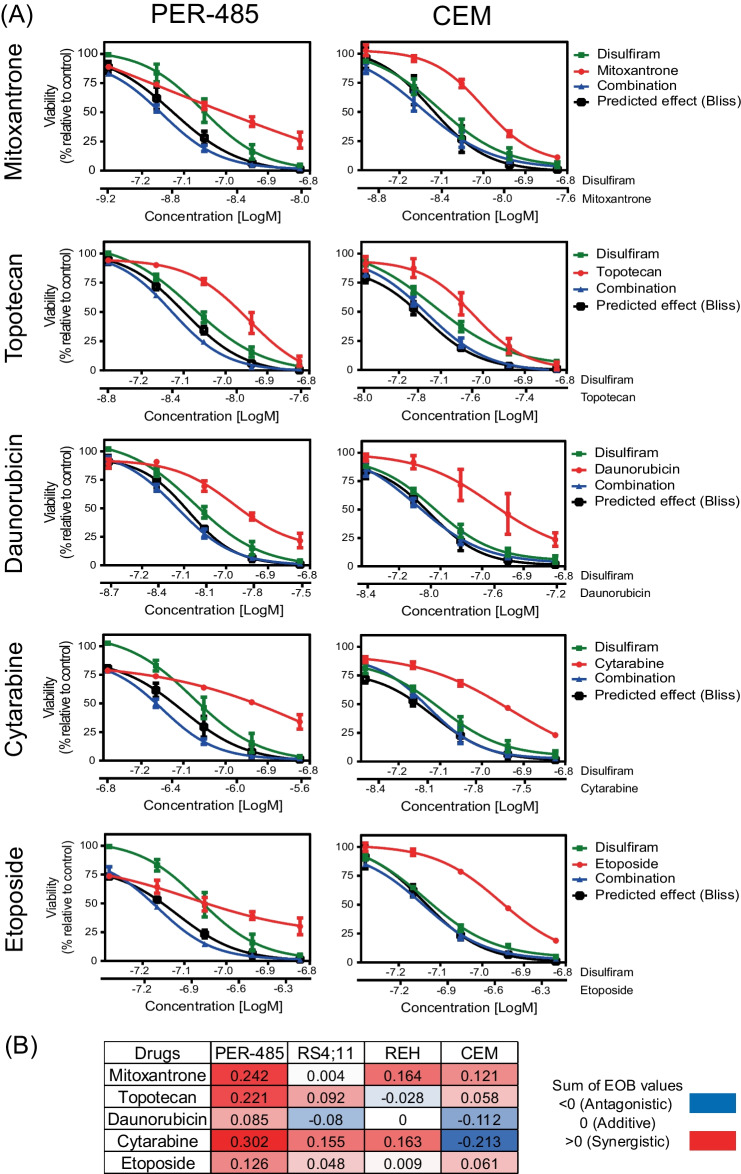
Disulfiram synergizes with conventional chemotherapeutic agents. **A** PER-485 and CEM leukemia cells were incubated with increasing concentrations of disulfiram alone, mitoxantrone, daunorubicin, etoposide, cytarabine, or topotecan alone or in combination at a fixed-ratio for 72 h, and viability was assessed using resazurin reduction-based cytotoxicity assays. The results are expressed as the mean viability (% relative to control) ± SE of three independent experiments. Synergy was assessed according to the Bliss Independence model. The black curve in each graph represents the predicted response based on an additive effect between the drugs. When the observed effect of the drug combination (curve in blue) runs below the black predicted effect curve, synergy occurs between the drugs. **B** Table displays mean Excess over Bliss (EOB) Sums that were calculated by adding up the difference between the actual fraction of cells affected by the drug combination and the expected fraction affected according to Bliss, at each dose combination. EOB $\text{>}$ 0 corresponds to a synergistic effect while EOB = 0 represents additivity and EOB < 0 antagonism

Progression of disulfiram into clinical trials for patients with leukemia will require combination with current standard of care treatment. Previous studies showed that the potency of disulfiram can be enhanced in solid cancer models by combining it with other standard of care drugs including chemotherapy [20, 31, 32]. To assess synergistic effects between disulfiram and clinically used chemotherapeutics (daunorubicin, etoposide, cytarabine, mitoxantrone, and topotecan), we performed synergy assays with a panel of acute leukemia cell lines, including cell lines that are highly resistant to current chemotherapeutics (e.g., PER-485) [23]. Cells were treated with increasing doses of either drug alone or the drug combination and synergy was assessed according to the Bliss Independency model [16]. Synergy was defined when the measured leukemia cell viability upon combination of the drugs (blue curve) was lower than what was predicted based on additive effects between the drugs (black Bliss Prediction curve). Excess over Bliss (EOB) Sums were calculated for each drug combination across all cell lines with an EOB Sum greater than 0 corresponding to synergy, and a value of 0 or below 0 representing an additive or antagonistic effect, respectively [16, 17]. The disulfiram/mitoxantrone and disulfiram/etoposide combinations were synergistic in three out of four tested leukemia cell lines, and additive in the remaining cell line (Fig. 3A, B, Supplementary Fig. 7). Upon combination of disulfiram with topotecan, daunorubicin, or cytarabine, the results were more variable between cell lines. Synergy was observed for all tested disulfiram combinations in the highly chemo-resistant PER-485 cells, while disulfiram/daunorubicin and disulfiram/cytarabine combinations were antagonistic in the T-ALL CEM cell line (Fig. 3A, B, Supplementary Fig. 7). Overall, our data indicate that the addition of disulfiram to chemotherapies might allow for the use of lower doses of conventional chemotherapeutic agents to achieve similar leukemia cell killing efficacy, but that effects are variable across leukemia subtypes and thus careful further preclinical evaluation is needed to identify markers that can predict response to these drug combinations.

**Fig. 4:**
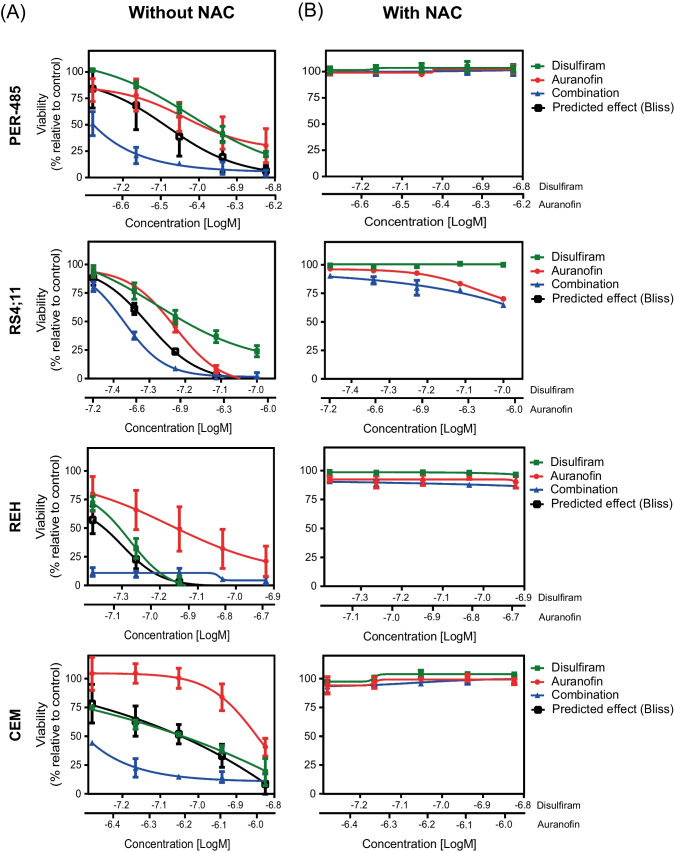
Disulfiram strongly synergizes with the FDA-approved drug auranofin. **A** PER-485, RS4;11, CEM, and REH cells were treated with increasing concentrations of disulfiram (DSF) alone, auranofin (AUR) alone, or the combination at a fixed-ratio for 72 h, and viability was assessed using resazurin reduction-based cytotoxicity assays. **B** Cells were pretreated with ROS scavenger NAC prior to addition of DSF, AUR, or the combination. Results are expressed as the mean viability (relative to control) ± SE of three independent experiments. Synergy was assessed according to the Bliss Independence model. The black curve in each graph represents the predicted response based on an additive effect between the drugs. When the observed effect of the drug combination (curve in blue) runs below the predicted effect curve, synergy occurs between the drugs

Studies in solid cancer models have reported a synergistic interaction between disulfiram and auranofin, a drug that is FDA-approved for the treatment of rheumatoid arthritis [33–35]. We identified auranofin as a candidate antileukemia agent in the same drug repurposing screen that yielded disulfiram [7]. In similar fashion to disulfiram, auranofin is very well-tolerated, not only in adults but also in children [36]. Given this suitability of auranofin to be considered for clinical trials for leukemia, we assessed the combinatorial effects of disulfiram and auranofin on acute leukemia cells. We observed very strong synergy between auranofin and disulfiram in all tested leukemia cell lines (PER-485, CEM, RS4;11, REH, Fig. 4A). This was further confirmed in 6 × 6 matrix synergy assays, demonstrating compelling pan-leukemic synergistic effects between the drugs at various drug dose combinations (Supplementary Fig. 8). Given our previous findings that the antileukemic effects of auranofin are also mediated through ROS induction, we next assessed whether the strong synergistic inhibitory effects of the disulfiram/auranofin combination on leukemia cell viability were mediated through ROS. Pretreatment with NAC abrogated the synergy between disulfiram and auranofin (Fig. 4B), confirming that increased ROS production is responsible for the antileukemic effect of the drug combination.

### Disulfiram inhibits the ex vivo viability of leukemia patient-derived xenograft cells

**Fig. 5:**
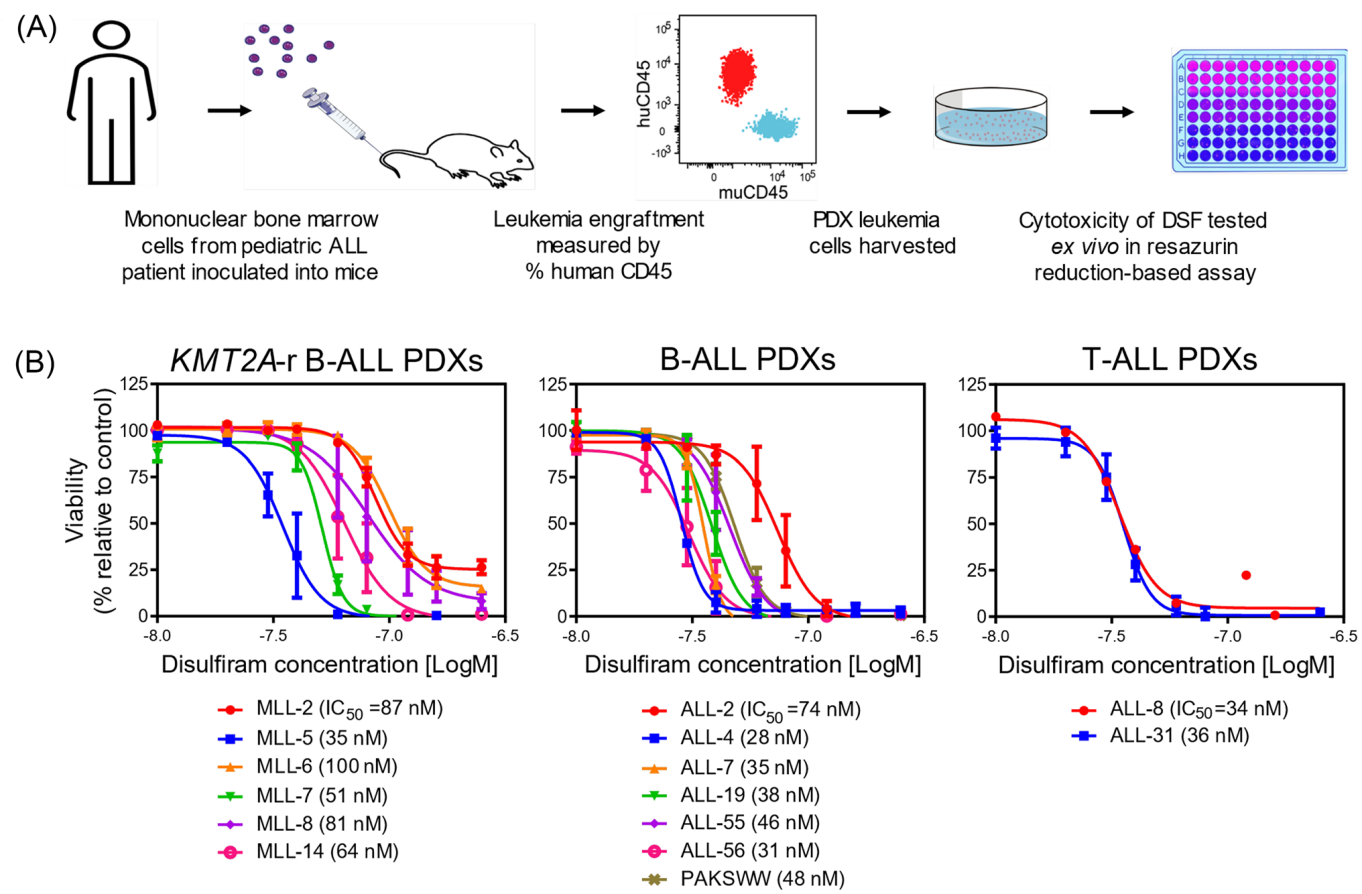
Cytotoxic effect of disulfiram against poor outcome and high-risk leukemia patient-derived xenograft (PDX) cells. **A** Schematic of the generation and ex vivo cytotoxicity testing of ALL PDX cells. **B** Full dose–response curves of disulfiram in *KMT2A*-r B-ALL, *KMT2A*-wt B-ALL, and T-ALL PDX cells after treatment with disulfiram and 100 nM copper for 72 h, as measured by resazurin reduction-based cytotoxicity assays. The results are expressed as the mean ± SE of three independent experiments

To assess the antileukemia activity of disulfiram in a more clinically relevant context, we explored the effects of the drug using a diverse panel of 15 PDXs established from children with high-risk or poor outcome acute leukemia. These PDX cells were previously generated by inoculating immunocompromised mice with patient bone marrow mononuclear cells (Fig. 5A) and were shown to be representative of the patients’ leukemias with respect to molecular phenotype and immunophenotype, as well as responsiveness to drugs [12, 14, 37–39]. The panel included PDX cells derived from infants with *KMT2A*-r ALL, Philadelphia chromosome-positive ALL (Ph + ALL), Ph-like ALL, and T-ALL and all but three (ALL-2, ALL-8, and ALL-19) were established from diagnostic samples obtained prior to treatment (Supplementary Table 2). Studying the PDX cells ex vivo, disulfiram exhibited similar cytotoxic effects against the PDX cells as against the established leukemia cell lines, with IC_50_ values ranging from 28 to 100 nM (Fig. 5B). Interestingly, the PDXs that were previously found to be less responsive in vivo to induction chemotherapy, comprising vincristine, dexamethasone, and l-asparaginase (VXL, MLL-5, ALL-4, and ALL-19) were among the most sensitive to disulfiram (Fig. 5B, Supplementary Table 2) [14]. Disulfiram sensitivity was not significantly different between *KMT2A*-r B-ALL, *KMT2A*-wt B-ALL, and T-ALL PDXs, even though a trend was observed for higher IC_50_ values for *KMT2A*-r versus *KMT2A*-wt B-ALL PDXs (Supplementary Fig. 9A). PDX cells established from diagnostic (pre-treatment) samples and from relapse (post-treatment) samples were equally sensitive to disulfiram (Fig. 5B, Supplementary Fig. 9B, Supplementary Table 2). Thus, disulfiram shows potent ex vivo activity against PDX cells derived from some of the most aggressive and treatment-resistant subtypes of leukemia, highlighting its therapeutic potential.

Our combined data, a priori, make a strong case for further evaluation of disulfiram as an antileukemia agent in in vivo models. However, we previously observed and reported that the plasma levels of disulfiram in mouse models are an order of magnitude lower compared to levels achieved in humans, indicating a substantially different pharmacokinetic profile of disulfiram in mice compared to humans, where concentrations that are effective in cell lines and PDX cells are difficult to be achieved in mice [8]. This precludes accurate in vivo preclinical testing of disulfiram using our current repertoire of PDX models in mice.

While survival rates for acute leukemia are increasing, many patients still succumb to the disease and the majority of those who survive suffer from the long-term detrimental side effects of current chemotherapeutic agents. The need for therapeutic strategies that are effective and more tolerable is evident. We here provide evidence that acute cell lines are sensitive to the cytotoxic effects of disulfiram and are more responsive to the drug than cell lines derived from solid tumors. Disulfiram exerts inhibitory effects on the viability and survival of xenograft cells derived from patients with the poorest outcome and treatment-resistant leukemia subtypes, induces rapid apoptosis in leukemia cells, and potentiates the effects of standard of care chemotherapeutic agents as well as the anti-rheumatoid arthritis drug auranofin.

Disulfiram is one of the oldest drugs in the therapeutic armamentarium, approved in 1948 by the FDA for the treatment of chronic alcoholism, and is thus highly characterized in relation to its tolerability, toxicity, and pharmacokinetic profile. The drug is well-tolerated, inexpensive, and orally administered. Its mechanism of action against cancer cells is multi-modal [11]. While the drug is currently under clinical investigation to assess its efficacy in patients with solid cancers, clinical trials in patients with leukemia are lacking. Our study provides evidence that leukemia cells are more sensitive to the anticancer activity of disulfiram than solid cancer cells, possibly based on their higher baseline levels of reactive oxygen species [7]. Overall, our study provides impetus to test disulfiram in clinical trials for patients with leukemia. Our observations of potent synergy between disulfiram and auranofin, another FDA-approved drug that is well-characterized and well-tolerated, further supports clinical testing of this drug combination.

### Supplementary Information

Below is the link to the electronic supplementary material.

Supplementary file1 (DOCX 3439 KB)

## Data Availability

Data are available from authors upon request.
