# Supplementary material for: FDA-approved disulfiram as a novel treatment for aggressive leukemia
Source: J Mol Med (Berl). 2024 Feb 13;102(4):507–19. doi: 10.1007/s00109-023-02414-4 (PMC10963497; doi:10.1007/s00109-023-02414-4)
Supplement: Supplementary file 1 — Supplementary file1 (DOCX 3439 KB) [file 109_2023_2414_MOESM1_ESM.docx]

**Supplementary Information**

**FDA-approved disulfiram as a novel treatment for aggressive leukemia**

**Mawar Karsa^1,2^, Lin Xiao^1,2^, Emma Ronca^1^, Angelika Bongers^1^, Dayna Spurling^1^, Ayu Karsa^1^, Sandra Catilena^3^, Anna Mariana^1,4^, Tim W Failes^1,2,4^, Greg M Arndt^1,2,4^, Laurence C Cheung^5,6,7^, Rishi S Kotecha^5,6,8,9^, Rosemary Sutton^1,2^, Richard B Lock^1,2,10^, Owen Williams^3^, Jasper de Boer^3^, Michelle Haber^1,2^, Murray D Norris^1,2,10^, Michelle J Henderson^1,2^, Klaartje Somers^1,2*^**

^1^ Children’s Cancer Institute, Lowy Cancer Research Institute, UNSW Sydney, Sydney, NSW, Australia

^2^ School of Clinical Medicine, UNSW Medicine & Health, UNSW Sydney, Sydney, NSW, Australia

^3^Cancer Section, Development Biology and Cancer Programme, UCL GOS Institute of Child Health, London, UK

^4^ ACRF Drug Discovery Centre for Childhood Cancer, Children’s Cancer Institute, Lowy Cancer Research Centre, UNSW Sydney, Sydney, NSW, Australia

^5^ Leukemia Translational Research Laboratory, Telethon Kids Cancer Centre, Telethon Kids Institute, Perth, WA, Australia

^6^ Curtin Medical School, Curtin University, Perth, WA, Australia

^7^ Curtin Health Innovation Research Institute, Curtin University, Perth, WA, Australia

^8^ Department of Clinical Haematology, Oncology, Blood and Marrow Transplantation, Perth Children’s Hospital, Perth, WA, Australia

^9^ Division of Paediatrics, School of Medicine, University of Western Australia, Perth, WA, Australia

^10^ UNSW Centre for Childhood Cancer Research, UNSW Sydney, Sydney, Australia

**Supplementary Figures**

**
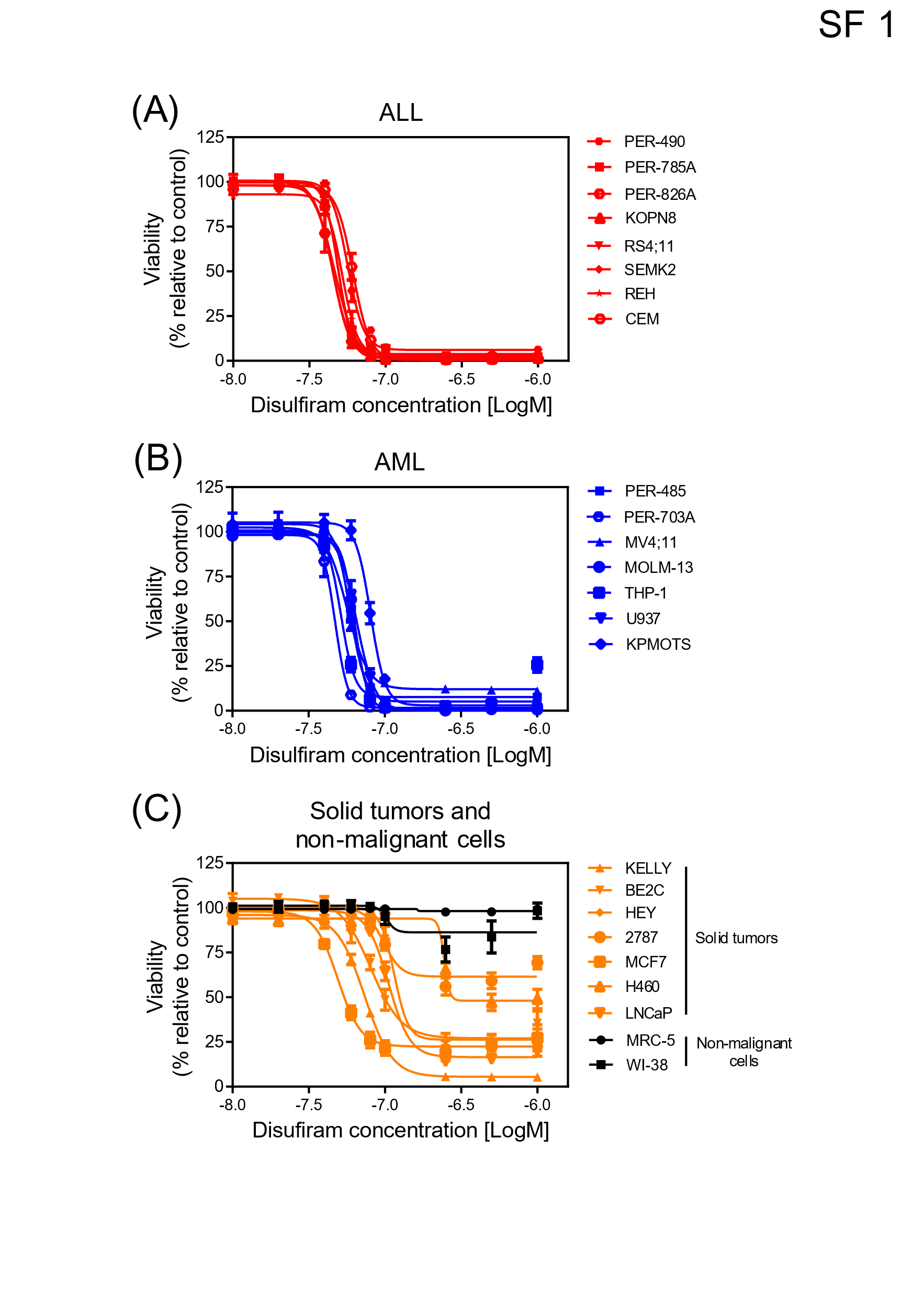
**

**Supplementary Figure 1: Cytotoxic activity of disulfiram.** Full dose-response viability curves of disulfiram in (A) ALL, (B) AML/MPAL, (C) solid tumor and non-malignant cell lines after a 72-hour treatment, as measured in resazurin reduction-based cytotoxicity assays. Viability percentages are expressed relative to control cells. The results are expressed as mean ± SE of three independent experiments.

**
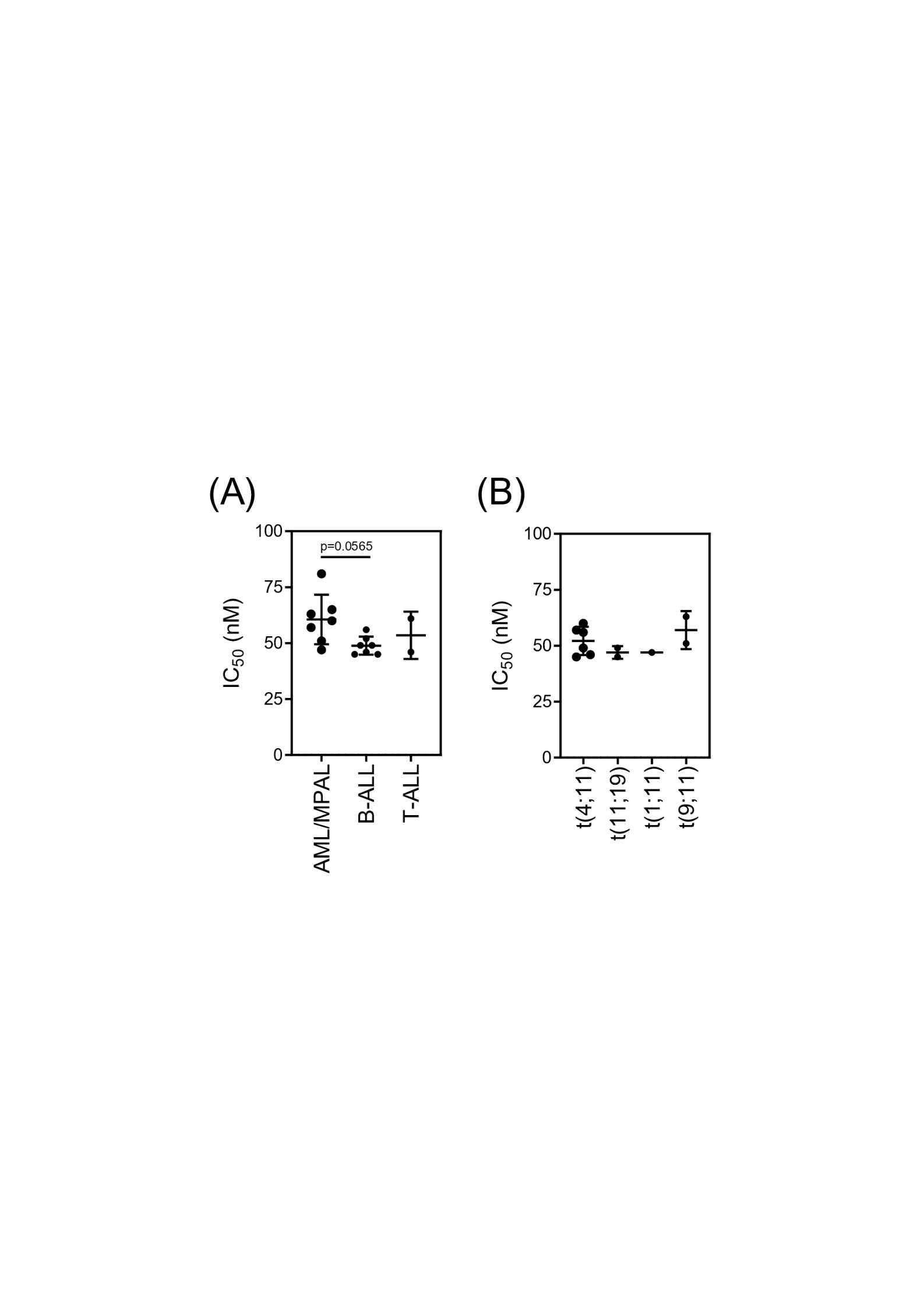
**

**Supplementary Figure 2: No significant associations between leukemia subtype and sensitivity to disulfiram.** Each dot represents the mean disulfiram IC_50_ value for a leukemia cell line as summarized in Table 1. (A) Comparison of disulfiram IC_50_ values between AML/MPAL, B-ALL and T-ALL cell lines. (B) Comparison of disulfiram IC_50_ values between *KMT2A*-r leukemia cell lines with different chromosomal translocations. Mean IC_50_ values were compared between groups by one-way ANOVA with Dunn’s correction for multiple comparisons.

**
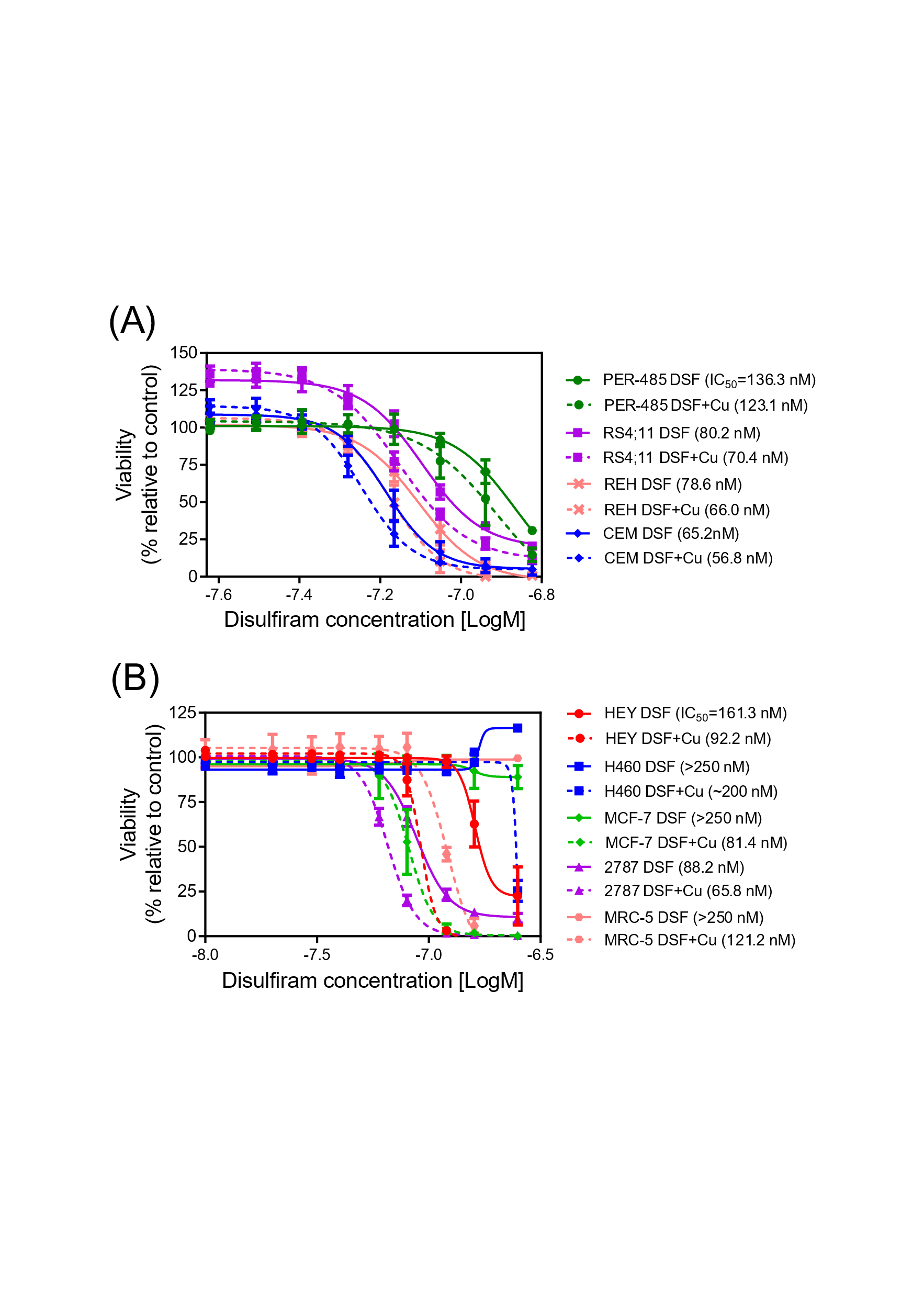
**

**Supplementary Figure 3: The addition of copper to disulfiram increases its cytotoxic effects.** (A) Comparison of the sensitivity of PER-485, RS4;11, REH and CEM leukemia cell lines to disulfiram without (DSF) or with 100 nM added copper (DSF+Cu) after a 72-hour treatment, as measured in resazurin reduction-based cytotoxicity assays. (B) Effect of addition of 100 nM copper to disulfiram on the viability of solid cancer and non-malignant cell lines after a 72-hour treatment, as measured in resazurin reduction-based cytotoxicity assays. The results in (A) and (B) are expressed as the mean ± SE of three independent experiments. The inhibitory concentration resulting in 50% reduction of cell survival relative to control (IC_50_) values were calculated by non-linear regression.


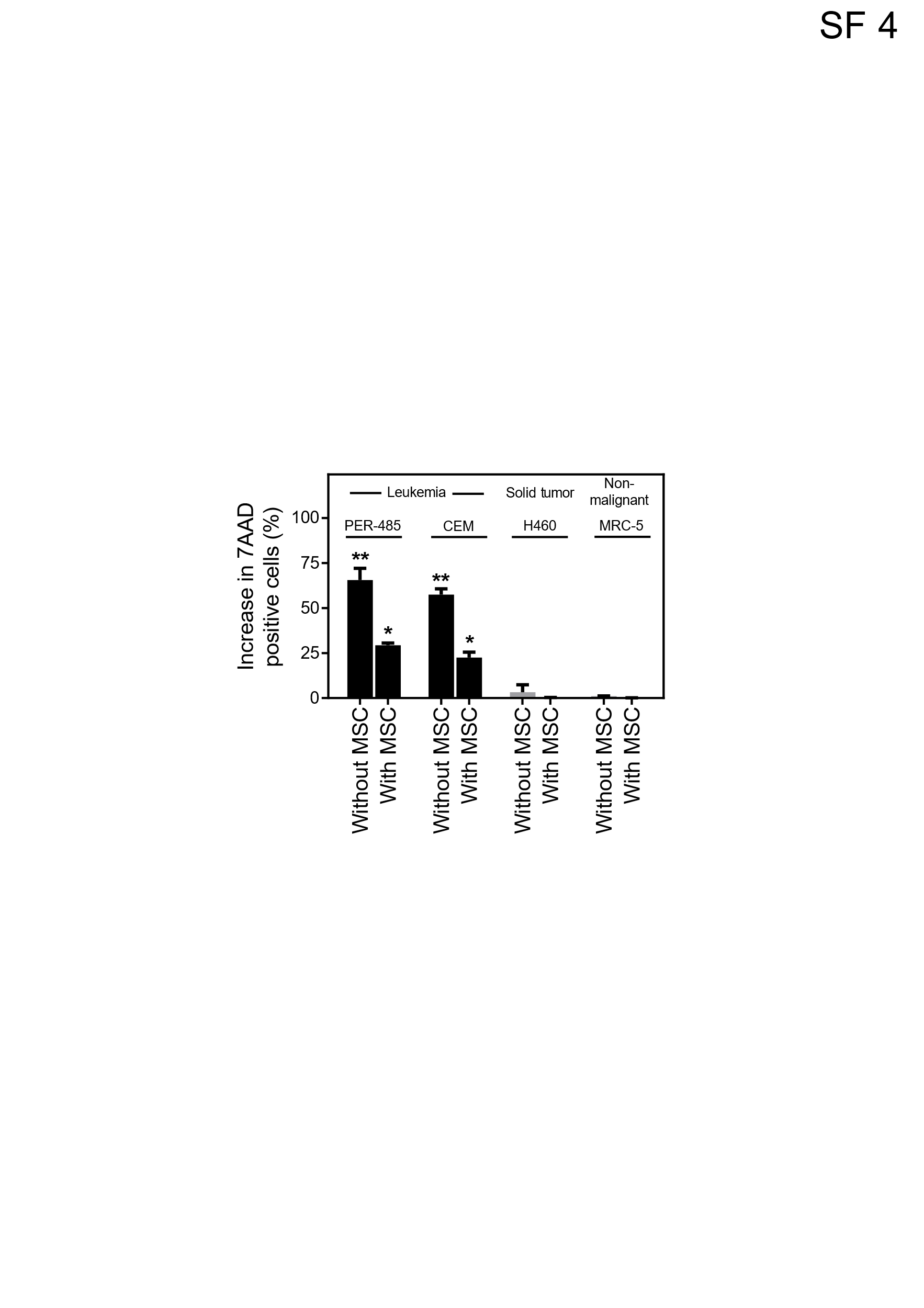


**Supplementary Figure 4: Disulfiram selectively kills leukemia cells in co-culture with MSCs.** Leukemia (PER-485, CEM), solid cancer (H460) and non-malignant (MRC-5) cells were added to MSC-lined (With MSC) or empty (Without MSC) wells and treated with 200 nM disulfiram for 24h after which the proportion of 7AAD-positive cells was determined by flow cytometry. Bars represent the increase in the % of 7AAD-positive cells relative to untreated cells (0%). One-sample t-tests were performed to assess the statistical significance of the increase in % 7AAD-positive cells after treatment compared to untreated cells. The results are displayed as mean ± SE of at least two independent experiments. Asterisks represent p-values. *, p<0.05; **, p<0.01.

**
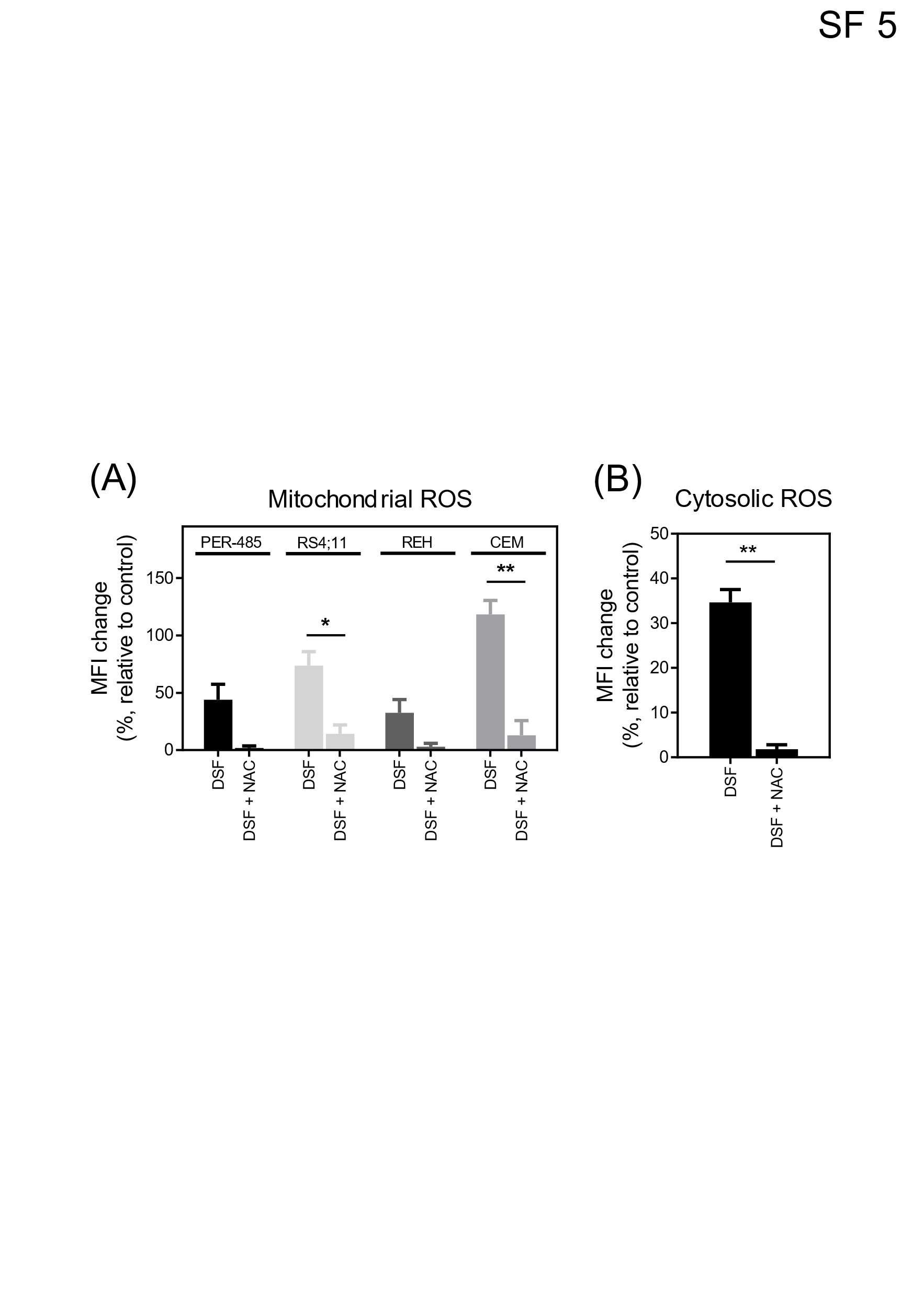
**

**Supplementary Figure 5: Pre-treatment with ROS scavenger NAC prevents disulfiram-induced ROS production in leukemia cells.** Impact of N-acetyl cysteine (NAC) on (A) mitochondrial and (B) cytosolic ROS levels as measured by flow cytometric analysis of MitoSOX and DCFDA, respectively, after a six-hour disulfiram (DSF) treatment (100 nM). Results are expressed as the mean ± SE of at least two independent experiments. ROS levels are expressed as percentage change in MFI relative to the MFI of control cells. Means between DSF and NAC+DSF treated cells were compared by paired t-tests. Asterisks represent p-values. *, p<0.05; **, p<0.01.


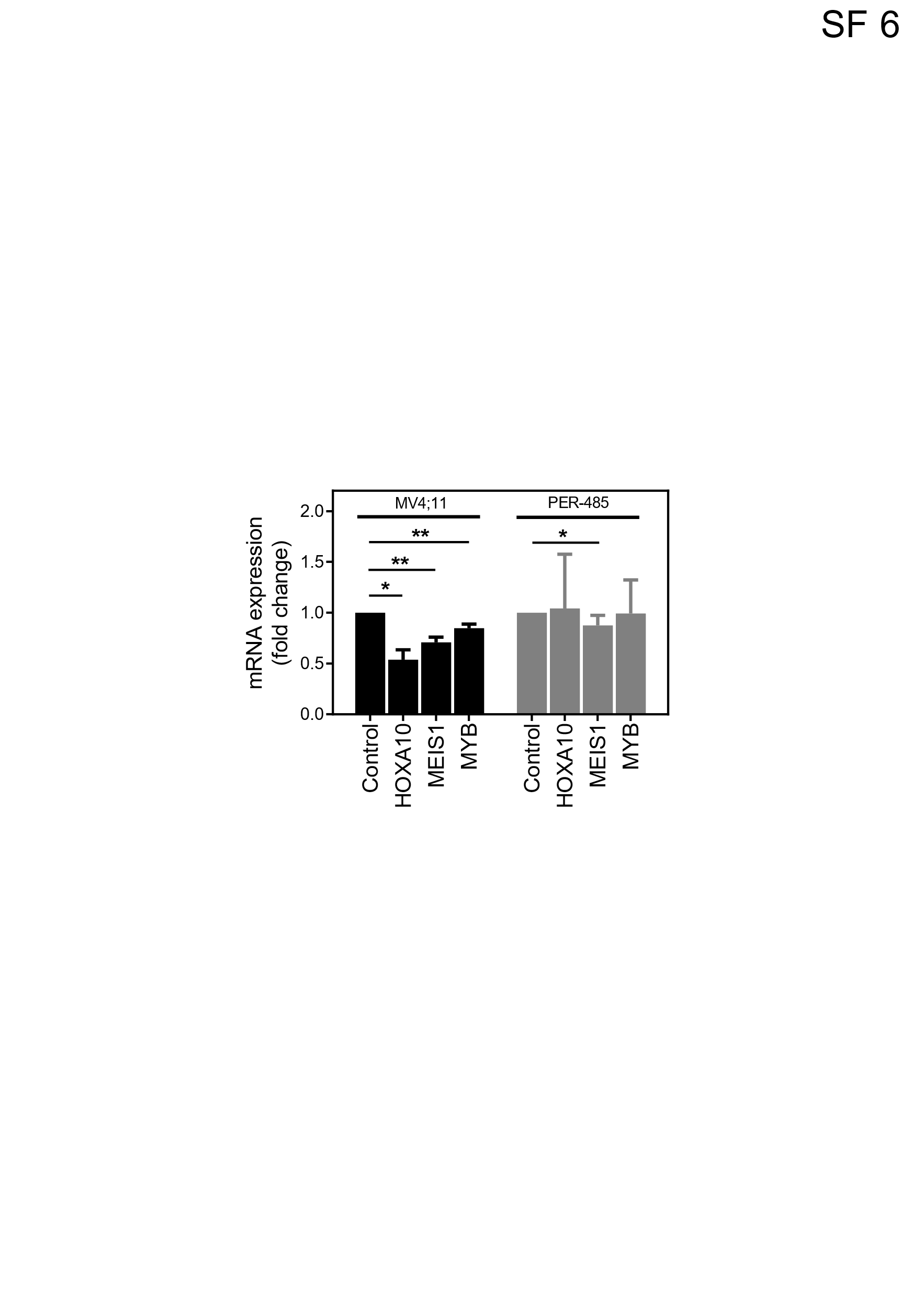


**Supplementary Figure 6:** **Disulfiram is able to decrease MLL fusion protein target gene expression in *KMT2A*-r leukemia cells.** mRNA expression level of MLL target genes *HOXA10*, *MEIS1* and *MYB* in *KMT2A*-r MV4;11 and PER-485 leukemia cell lines after DSF (0.4 µM and 1.0 µM, respectively) treatment for four hours relative to control cells. One-sample t-tests were used to assess the significance of gene expression changes upon treatment. Asterisks represent p-values. *, p<0.05; **, p<0.01; ***, p<0.001.


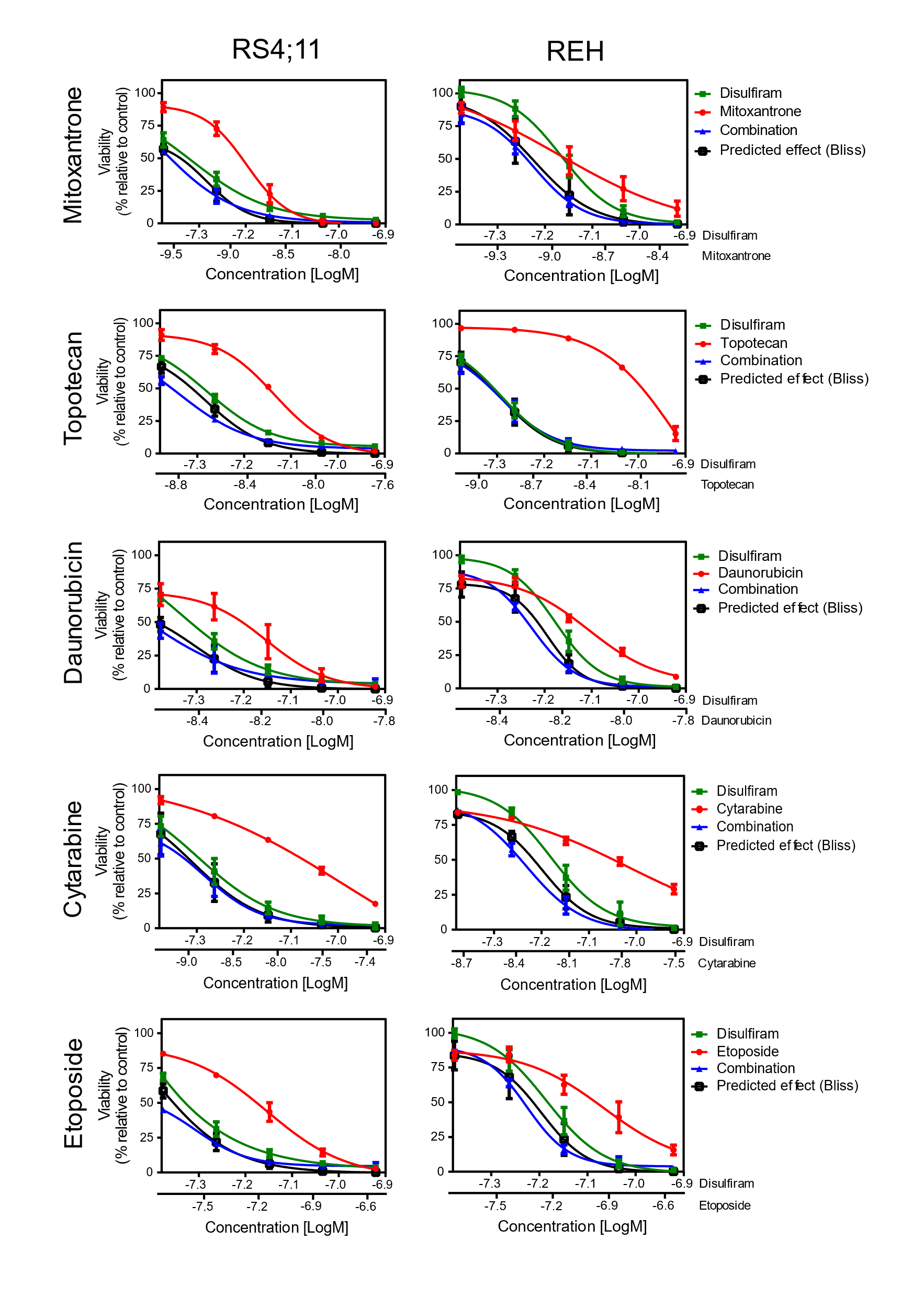


**Supplementary Figure 7:** **Disulfiram synergizes with conventional chemotherapeutic agents**. RS4;11 and REH leukemia cells were incubated with increasing concentrations of disulfiram alone, mitoxantrone, daunorubicin, etoposide, cytarabine or topotecan alone or in combination at a fixed-ratio for 72 hours, and viability was assessed using resazurin reduction-based cytotoxicity assays. The results are expressed as the mean viability (relative to control) ± SE of three independent experiments. Synergy was assessed according to the Bliss Independence model. The black curve in each graph represents the predicted response based on the additive effect of both drugs. When the observed effect of the drug combination (curve in blue) runs below the black predicted effect curve, synergy occurs between the drugs.


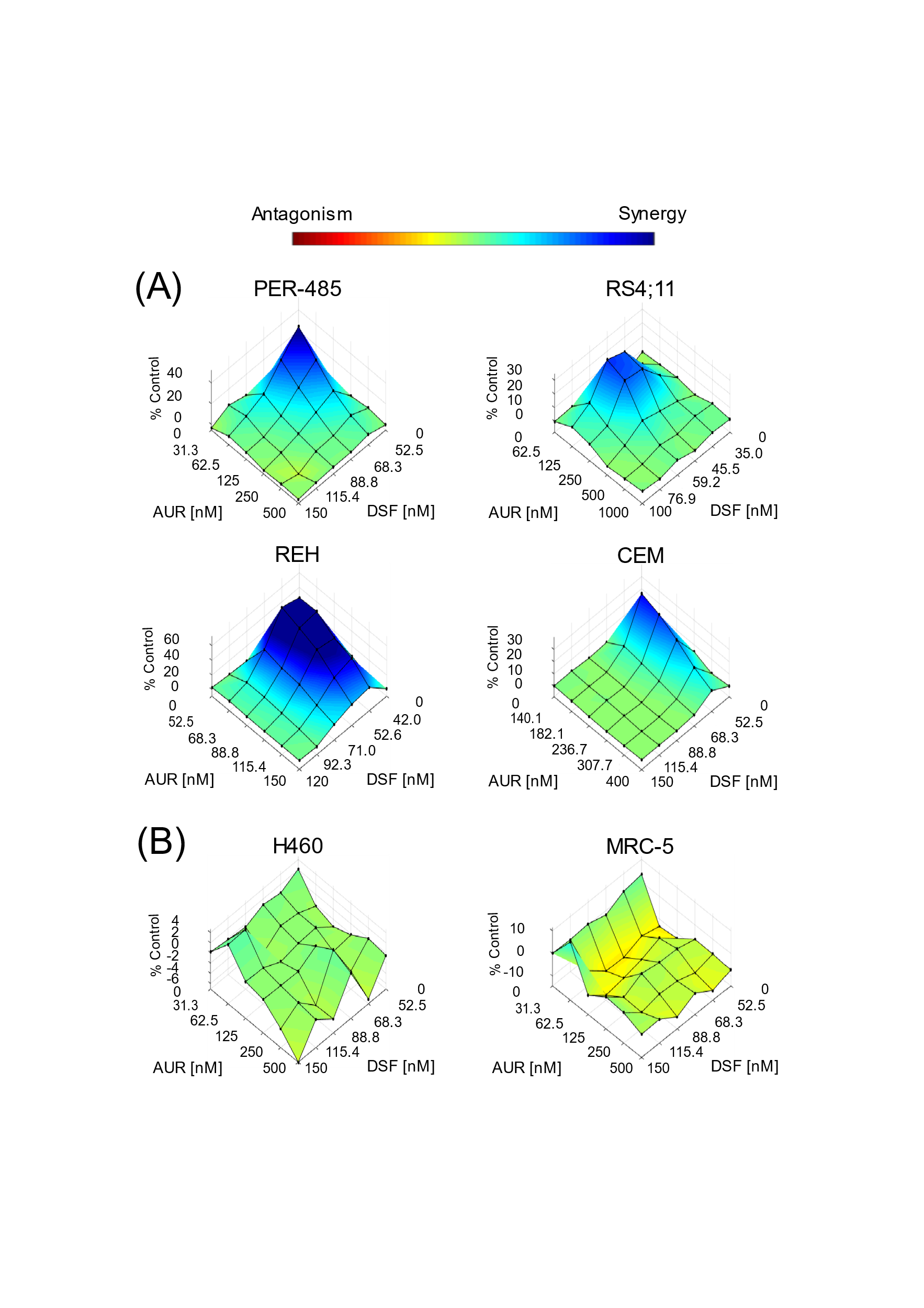


**Supplementary Figure 8:** **Disulfiram strongly synergizes with auranofin**. (A) Leukemia cell lines (PER-485, RS4;11, REH, CEM), and (B) a solid cancer cell line (H460) and non-malignant cells (MRC-5) were treated with disulfiram (DSF) combined with auranofin (AUR) in a 6×6 matrix with incremental increases in drug concentrations. Cell viability was measured by resazurin reduction-based assays at 72 hours. Data were visualized with Combenefit software. The plots display the synergy distribution according to the Bliss Independence model. Representative plots of at least two independent runs are shown.

**
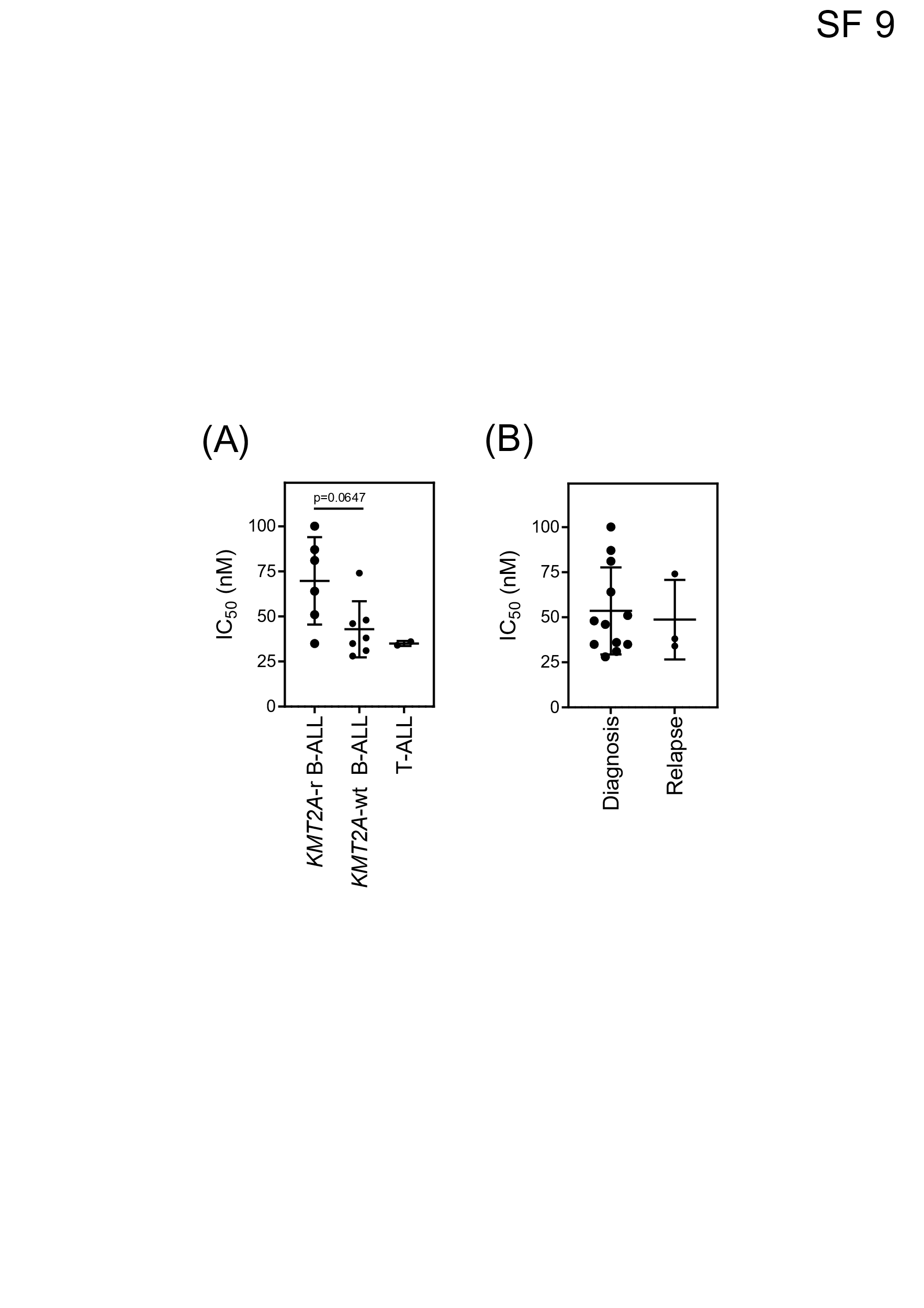
**

**Supplementary Figure 9:** **No significant associations between PDX leukemia subtype and sensitivity to disulfiram.** Plots show mean disulfiram IC_50_ value for each PDX as displayed in Figure 5. A) Comparison of disulfiram IC_50_ values between *KMT2A*-r B-ALL, *KMT2A*-wt B-ALL and T-ALL PDXs. B) Comparison of disulfiram IC_50_ values between PDXs established from diagnostic versus relapse samples. Mean IC_50_ values were compared between groups by one-way ANOVA with Dunn’s correction for multiple comparisons.

**Supplementary Tables**

**Supplementary Table 1: Characteristics of cell lines used in this study**

|  | **Cell Line** | **Disease** | **Disulfiram IC_50_ (nM)** | **Supplier/Source** |
| --- | --- | --- | --- | --- |
|  |  |  |  |  |
| **Leukemia cells** | | |  |  |
| B-ALL | PER-485 (RRID:CVCL_W984) | Infant *KMT2A*-r Mixed Phenotype Acute Leukemia | 60 | Kotecha R and Cheung L, Telethon Kids Institute, Perth, AU |
|  | PER-703 (RRID:CVCL_IY99) | Infant *KMT2A*-r Mixed Phenotype Acute Leukemia | 47 | Kotecha R and Cheung L, Telethon Kids Institute, Perth, AU |
|  | PER-826 (RRID:CVCL_IZ05) | Infant *KMT2A*-r ALL | 45 | Kotecha R and Cheung L, Telethon Kids Institute, Perth, AU |
|  | PER-490 (RRID:CVCL_W986) | Infant *KMT2A*-r ALL | 56 | Kotecha R and Cheung L, Telethon Kids Institute, Perth, AU |
|  | PER-785 (RRID:CVCL_IZ03) | Infant *KMT2A*-r ALL | 49 | Kotecha R and Cheung L, Telethon Kids Institute, Perth, AU |
|  | KOPN-8 (RRID:CVCL_1866) | Infant *KMT2A*-r ALL | 45 | DSMZ (ACC 552) |
|  | RS4;11 (RRID:CVCL_0093 | *KMT2A*-r Pre-B cell ALL | 46 | ATCC (ATCC^®^ CRL-1873^™^) |
|  | SEMK2 (RRID:CVCL_S906) | *KMT2A*-r Pre-B cell ALL | 49 | Armstrong S, Memorial Sloan Kettering Cancer Center, New York, USA |
|  | REH (RRID:CVCL_1650) | TEL-ALM1 translocated pre-B cell ALL | 52 | ATCC (ATCC^®^ CRL-8286^™^) |
| T-ALL | CCRF-CEM (RRID:CVCL_0207) | T-ALL | 61 | ATCC (ATCC^®^ CRM-CCL-119^™^) |
|  | Jurkat (RRID:CVCL_0065) | T-ALL | 58 | ATCC (ATCC® TIB-152™) |
| AML | THP-1 (RRID:CVCL_0006) | Infant *KMT2A*-r AML | 51 | W Jessup, Centre for Vascular Research, NSW, Australia |
|  | MV4;11 (RRID:CVCL_0064) | *KMT2A*-r AML | 57 | ATCC (ATCC^®^ CRL-9591^™^) |
|  | MOLM13 (RRID:CVCL_2119) | *KMT2A*-r AML | 63 | R D’Andrea, IMVS, South Australia, Australia |
|  | U937 (RRID:CVCL_0007) | *CALM-AF10* translocated AML | 65 | ATCC (ATCC^®^ CRL-1593.2^™^) |
|  | KP-MO-TS (RRID:CVCL_7985) | *CALM-AF10* translocated AML | 81 | Imamura T, Kyoto Prefectural University of Medicine, Kyoto, Japan |
| **Solid tumor cells** | |  |  |  |
|  | KELLY (RRID:CVCL_2092) | Neuroblastoma | 73 | ECACC (92110411) |
|  | BE(2)-C (RRIC:CVCL_0529) | Neuroblastoma | 81 | J Biedler, Memorial Sloan-Kettering Cancer Centre, NY, USA |
|  | HEY (RRID:CVCL_0297) | Ovarian cancer | 115 | G Chenevix-Trench, QIMR Berghofer Medical Research Institute, QLD, Australia |
|  | 2787 (RRID not applicable) | Endometrial ovarian cancer | 49 | A de Fazio; Westmead Millennium Institute for Medical Research, NSW, Australia |
|  | MCF-7 (RRID: CVCL_0031) | Breast adenocarcinoma | >1000 | ATCC (ATCC^®^ HTB-22^™^) |
|  | H460 (RRID:CVCL_0459) | Lung carcinoma | 500 | ATCC (ATCC^®^ HTB-177^™^) |
|  | LNCaP (RRID:CVCL_0395) | Prostate carcinoma | 106 | ATCC (ATCC^®^ CRL-1740^™^) |
| **Non-malignant cells** | |  |  |  |
|  | MRC-5 (RRID:CVCL_0440) | Non-malignant lung fibroblast | >1000 | ATCC (ATCC^®^ CCL-171^™^) |
|  | WI-38 (CVCL_0579) | Non-malignant lung fibroblast | >1000 | ATCC (ATCC^®^ CCL-75^™^) |

**Supplementary Table 2: Patient demographics and key molecular lesions of the pediatric ALL PDXs used in the study**

| **PDX** | **ALL subtype** | **Age at diagnosis (years)** | **Sex** | **Disease status at biopsy** | **Structural variations** | **Copy number variations** | **Single nucleotide variants, gene (amino acid change, variant allele frequency)** | **ORM score to VXL treatment** |
| --- | --- | --- | --- | --- | --- | --- | --- | --- |
| **MLL-2** | *KMT2A*-r ALL | <1 | M | Diagnosis | KMT2A:AFF1; DUX4 SV | ND | EYS (TQ3100fs, 0.45); TCF3 (T12A, 0.42); MGA (K1086N, 0.39); | MCR |
| **MLL-5** | *KMT2A*-r ALL | <1 | M | Diagnosis | KMT2A:MLLT10; PAX5:ZCCHC7 | ND | ND | PD2 |
| **MLL-6** | *KMT2A*-r ALL | <1 | M | Diagnosis | KMT2A:MLLT1; MEF2D:PTMA | ND | FBN2 (H2694Q, 0.51); BRCA2 (N2135del, 0.48); CROCC (G1262R, 0.48); PHLPP1 (K1577E, 0.52); | MCR |
| **MLL-7** | *KMT2A*-r ALL | <1 | M | Diagnosis | KMT2A:AFF1; MEF2D:PTMA; KMT2A:PTPRC | ND | FLG (E3292G, 0.5); FLT3 (I836del, 0.46); MET (L575I, 0.47); CHEK1 (I465T, 0.54); CSF1R (S422C, 0.54); | MCR |
| **MLL-8** | *KMT2A*-r ALL | <1 | F | Diagnosis | KMT2A:MLLT1 | ND | KMT2D (P1964R, 0.46); KIAA1549 (G1633S, 0.15); EPHA3 (L10F, 0.34); CREBBP (H854P, 0.57); SMC3 (S39R, 0.19); KMT2E (P1545L, 0.23); MTAP (R252Q, 0.52); | MCR |
| **MLL-14** | *KMT2A*-r ALL | <1 | F | Diagnosis | KMT2A:MLLT1 | ND | LRIG1 (L288F, 0.46); KMT2D (P2301S, 0.51); KMT2D (R5007W, 0.46); MYH11 (L1004V, 0.52); ATR (L2076V, 0.55); | CR |
| **ALL-2** | BCP ALL | 5.4 | F | Relapse | ND | CDKN2A (HOMDEL), CDKN2B (HOMDEL) | FLT3 (Y572S, 0.62); LZTR1 (F258fs, 0.45); KMT2D (G2863fs, 0.27); KMT2D (G2863fs, 0.45); PTPRC (H1241Y, 0.25); NT5C2 (R238W, 0.45); CREBBP (D1481H, 0.59); MLLT10 (P253S, 0.71); TSPYL2 (P686A, 0.46); | MCR |
| **ALL-7** | BCP ALL | 7.3 | M | Diagnosis | TCF3:HLF | ND | FYN (T78R, 0.41); KRAS (G12V, 0.43); EYS (S1490L, 0.56); | MCR |
| **ALL-19** | BCP ALL | 16.1 | M | Relapse | NUP214:ABL1 | CDKN2A (HOMDEL), IKZF1 (HOMDEL) | MLLT4 (P443R, 0.47); KRAS (L23R, 0.54); GNB1 (89_90insD, 0.49); FLG (S1848F, 0.56); | PR |
| **ALL-4** | Ph+ ALL | 8.7 | M | Diagnosis | BCR:ABL1 | IKZF1 (DEL) | MLLT10 (A846G, 0.58); DDX3X (T323N, 0.51); KBTBD4 (E207fs, 0.38); BCR (E552G, 0.3); FGF2 (N113S, 0.55); | PD2 |
| **ALL-55** | Ph+ ALL | 14.5 | M | Diagnosis | BCR:ABL1 | ND | LRIG1 (Q1053L, 0.43); CTNNB1 (N287S, 0.48); KMT2D (T4350A, 0.47); ABL2 (G896R, 0.63); GIGYF2 (E875del, 0.47); | - |
| **ALL-56** | Ph+ ALL | 10 | M | Diagnosis | BCR:ABL1 | ND | ND | - |
| **PAKSWW** | Ph-like ALL | 15.1 | M | Diagnosis | ND | ND | KRAS (G12V, 0.47); PIK3CG (R273H, 0.2); TP53 (R248Q, 0.99); TSC1 (P56L, 0.45); SMC1A (R57W, 0.99); | - |
| **ALL-8** | T-ALL | 12.6 | M | Relapse | ND | CDKN2A (HOMDEL), CDKN2B (HOMDEL) | FAT2 (R2678*, 0.43); NT5C2 (R367Q, 0.56); FBXW7 (R465C, 0.42); ASXL1 (D863G, 0.47); SMARCA4 (R1189Q, 0.49); IL1B (R75K, 0.47); FBN2 (D1008H, 0.51) | MCR |
| **ALL-31** | T-ALL | 10.1 | M | Diagnosis | PTMA:NPM1; LMO1:TRBC1; LMO1:TRBC2 | CDKN2A (HOMDEL), CDKN2B (HOMDEL) | NRG2 (A792T, 0.49); MLLT4 (R915H, 0.47) | - |
| BCP, B-cell precursor; CR, complete response; DEL, shallow deletion; del, deletion; fs, frameshift mutation; HOMDEL, homozygous deletion; ins, insertional mutation; MCR, maintained complete response; ND, not detected; ORM, objective response measure; PD2, progressive disease 2; PR, partial response; VXL, combination of vincristine, dexamethasone and *L*-asparaginase; *, nonsense mutation | | | | | | | | |
